# The Rubber Nuisance

**Published:** 1870-01

**Authors:** 


### THE RUBBER NUISANCE.

WE are often asked if we think the Vulcanite Company, *alias* Josiah Bacon & Co., will try to enforce the claims of the Cumming's patent after the expiration of the Goodyear. The profession will not be so stupid as to need the rubber by that time, and we hope not so unprincipled as to use a material so objectionable, now that its bad properties are becoming understood. A well posted Dentist could not afford to pay Mr. Bacon, or the Company, fifteen cents for an office right for the full term of both patents.

But what shall we use says one ? The question may be asked by genuine Dentists who feel that their patrons will demand something low priced. It is often asked by those who can mold rubber plates, but know nothing about true Dentistry. An old farmer was trying to get through the lines, after a battle, to see his sons. He was asked if he was a physician. "Well," said he, "they don't exactly call me doctor at home ; but I can make a leetle the saftest and healin'est salve you most ever seed." To those like the salve man we have nothing to say ; but to the others we say, if you must do low priced work, and we suppose that is necessary, you can do better for your patient and yourself, by wholly abandoning rubber.

Almost any metallic plate in use is far preferable to vulcanite. The old cheoplastic work is far better. The "adamantine base," and "Weston's metal" are probably improvements on this. We can make any number of metallic compounds better than rubber, and which may be as readily used. Starr's patent, which is aluminum plate, the teeth being united to it by solder, is cleaner, lighter, brighter and healthier than rubber, and quite as durable and more easily repaired. And the office right for the full term of the patent is $25, instead of $50 a year, as with Josiah Bacon & Co.

Then, a recent improvement, not patented, has made its appearance from the office of Watt & Williams, Xenia, O. It consists in preparing an alloy with electric properties adapted to the plate on which it is to be used, and which may be used on aluminum plate, or on platinized silver plate, now being prepared by George Watt & Co. The plate is swaged, and the teeth, in sections, are attached by the alloy. It can be made in about the same time as rubber work, is more cleanly, is stronger, neater and more healthy. The metal being a good conductor of heat and electricity, and containing no poisonous compounds of mercury, is free from the objections which lie against the vulcanite.

Any good Dentist can make this work with a little instruction. We presume an arrangement can be made with Dr. Williams by which those needing and desiring such instruction can obtain it. With this and the Star work for low prices; with gold and platinum for more thorough operations, we may let the Company take their rubber and Bacon to the dry heat vulcanizer we read about.

